# Analytical and clinical validation of a custom 15-gene next-generation sequencing panel for the evaluation of circulating tumor DNA mutations in patients with advanced non-small-cell lung cancer

**DOI:** 10.1371/journal.pone.0276161

**Published:** 2022-10-18

**Authors:** Yock Ping Chow, Norziha Zainul Abidin, Ken Siong Kow, Lye Mun Tho, Chieh Lee Wong

**Affiliations:** 1 Clinical Research Centre, Sunway Medical Centre, Petaling Jaya, Selangor Darul Ehsan, Malaysia; 2 Molecular Diagnostics Laboratory, Sunway Medical Centre, Petaling Jaya, Selangor Darul Ehsan, Malaysia; 3 Department of Medicine, Sunway Medical Centre, Petaling Jaya, Selangor Darul Ehsan, Malaysia; 4 Haematology Unit, Department of Medicine, Sunway Medical Centre, Petaling Jaya, Selangor Darul Ehsan, Malaysia; 5 Centre for Haematology, Hammersmith Hospital, London, United Kingdom; 6 Faculty of Medicine, Imperial College London, London, United Kingdom; Osmania University, Hyderabad, India, INDIA

## Abstract

**Background:**

This is a pilot proof-of-concept study to evaluate the utility of a custom 15-gene circulating tumor DNA (ctDNA) panel as a potential companion molecular next-generation sequencing (NGS) assay for identifying somatic single nucleotide variants and indels in non-small-cell lung cancer (NSCLC) patients. The custom panel covers the hotspot mutations in *EGFR*, *KRAS*, *NRAS*, *BRAF*, *PIK3CA*, *ERBB2*, *MET*, *KIT*, *PDGFRA*, *ALK*, *ROS1*, *RET*, *NTRK1*, *NTRK2* and *NTRK3* genes which serve as biomarkers for guiding treatment decisions in NSCLC patients.

**Method:**

The custom 15-gene ctDNA NGS panel was designed using ArcherDX Assay Designer. A total of 20 ng or 50 ng input ctDNA was used to construct the libraries. The analytical performance was evaluated using reference standards at different allellic frequencies (0.1%, 1%, 5% and parental). The clinical performance was evaluated using plasma samples collected from 10 treatment naïve advanced stage III or IV NSCLC patients who were tested for tissue *EGFR* mutations. The bioinformatics analysis was performed using the proprietary Archer Analysis Software.

**Results:**

For the analytical validation, we achieved 100% sensitivity and specificity for the detection of known mutations in the reference standards. The limit of detection was 1% allelic frequency. Clinical validation showed that the clinical sensitivity and specificity of the assay for detecting *EGFR* mutation were 83.3% and 100% respectively. In addition, the NGS panel also detected other mutations of uncertain significance in 6 out of 10 patients.

**Conclusion:**

This preliminary analysis showed that the custom 15-gene ctDNA NGS panel demonstrated good analytical and clinical performances for the *EGFR* mutation. Further studies incorporating the validation of other candidate gene mutations are warranted.

## Introduction

Non-small cell lung cancer (NSCLC) represents the most common type of lung cancer which accounts for approximately 11% of all cancer types in Malaysia [1]. Nearly 90% of lung cancer patients in Malaysia are diagnosed with stage III or IV disease, and have poor 5-year overall survival of about 8% [2]. While PD-1/CTLA-4 inhibitors are recommended as mainstay therapy for patients with overexpression of PD-L1 protein, the role of PD-L1 as a predictive biomarker of response remains suboptimal, as emerging evidences suggested that a subset of PD-L1 negative patients respond to the treatment [3]. As such, combination of mutational profiling and PD-L1 expression may serve as better predictive biomarkers of response and warrants further investigation [4].

In recent years, numerous targeted therapies have been approved by US Food and Drug Administration (FDA) for treating NSCLC with actionable mutations, and have shown prolonged survival. These include gefitinib, ertlotinib, or osimertinib for *EGFR* mutations; combination of dabrafenib and trametinib for *BRAF* mutations; crizotinib, alectinib and lorlatinib for *ALK*/*ROS* gene fusions; larotrectinib for *NTRK* gene fusions; and Enhertu for *ERBB2* mutations. To truly enable precision oncology, National Comprehensive Cancer Network (NCCN) and US FDA have recommended biomarker testing for genetic alterations in at least seven cancer genes (*EGFR*, *ALK*, *RET*, *ROS1*, *MET*, *BRAF*, and *NTRK*) for guiding treatment decisions in NSCLC [5–7]. Also, the consensus statement on molecular testing has been made available to guide the treatment strategy for NSCLC in Malaysia [8]. As the acquisition of hotspot DNA point mutations in the *ALK*, *RET*, *ROS1*, and *NTRK* genes confers drug resistance, molecular testing of these hotspot mutations in conjunction with fusion gene assessment via fluorescence in situ hybridization (FISH) or transcriptomic-based methods is crucial in the field of precision oncology [9]. As the number of approved targeted therapies continues to expand, it is therefore imperative to incorporate multigene panel testing as a routine companion diagnostic tool to broaden therapeutic options for cancer patients.

Tumor tissue biopsy is the gold standard for routine pathological assessment and molecular testing for lung cancer patients. However, performing a tissue biopsy can sometimes lead to complications, a single tumor biopsy may not truly represent a heterogenous tumor sample, and nearly 30% of the biopsied tissues are inaccessible or contain insufficient material to support biomarker testing [10, 11]. As such, noninvasive identification of tumor-associated mutations through body fluids such as blood represents an attractive alternative to tissue-based methods. In recent years, the advent of multiplex real-time PCR, droplet digital PCR and next-generation sequencing (NGS) have enabled detection of mutations in circulating tumor DNA (ctDNA). Typically, ctDNA are short fragment DNA (range 150–180 bp) released from apoptotic or necrotic tumor cells into the circulating blood and serve as surrogate markers for molecular oncology testing. Liquid biopsies not only allow molecular assessment to be performed in patients whose tissue biopsies are inaccessible or inadequate, but also allow detection of acquired drug-resistance mutations in refractory or relapsed patients and facilitate re-adjustment of treatment regimen upon disease progression and lead to improved treatment outcome [11–13].

Precision oncology has revolutionized cancer care as evidenced by the growing list of biomarker guided targeted therapies for solid tumors including NSCLC [14]. It is therefore of clinical importance to develop a robust and affordable molecular diagnostic tool that is capable of screening multiple gene mutations in a single assay. Molecular testing of multiple gene targets becomes more cost-effective and time efficient with NGS than sequential testing with single-gene approaches. Although NGS has been validated as a promising approach to detect mutations in formalin-fixed paraffin embedded (FFPE) NSCLC samples at reduced cost and faster turnaround time relative to single gene testing approaches [15], development of a robust assay for detecting mutations in ctDNA remains challenging. Some studies showed excellent concordance between mutations detected in tumor tissue and ctDNA (>80%) [16–20], whilst some studies found lack of concordance [21–23]. Within this context, our study aims to develop and validate the clinical utility of a custom 15-gene NGS panel for detecting clinically significant mutations in ctDNA in advanced stage III or IV NSCLC patients.

This custom 15-gene NGS panel covers the activating and drug resistance hotspot DNA point mutations and indels in *EGFR*, *KRAS*, *NRAS*, *BRAF*, *PIK3CA*, *ERBB2*, *MET*, *KIT*, *PDGFRA*, *ALK*, *ROS1*, *RET*, *NTRK1*, *NTRK2* and *NTRK3* genes which serve as biomarkers for guiding treatment decisions in NSCLC patients. The panel may also guide potential treatment with mobocertinib, amivantamab-vmjw (*EGFR* exon 20 insertion mutations), sotorasib (*KRAS* G12C), osimertinib (*EGFR* T790M), erlotinib, dacomitinib, gefitinib, afatinib, osimertinib (*EGFR* exon 19 deletion or exon 21 L858R), afatinib (*EGFR*: S768I, L861Q, and/or G719X), combination of dabrafenib and trametinib *(BRAF* V600E), tepotinib, capmatinib (*MET* exon 14 skipping alteration), and ado-trastuzumab emtansine, trastuzumab deruxtecan (*ERBB2* mutations). In addition, the NGS panel also covers secondary resistance mutations such as fusion mutations in *MET*, *RET*, *ALK*, *ROS1*, *NTRK1*, *NTRK2*, or *NTRK3* genes for which matched approved therapies are available. The selected hotspot mutations could be used to guide treatment with pralsetinib or selpercatinib targeting *RET* gene fusions; entrectinib or Larotrectinib targeting *NTRK*-positive tumors; crizotinib, ceritinib, lorlatinib, brigatinib, or alectinib targeting *ALK*-positive tumors; crizotinib, ceritinib, or lorlatinib targeting *ROS1*-positive tumors. The remaining genes mutations in *NRAS*, *KIT*, *PIK3CA*, and *PGFRA* have been observed in NSCLC patients [24–27] and are included in the NGS panel as exploratory predictive biomarkers.

## Materials and methods

### Ethics statement

The ethics approval for this study was obtained from Sunway Medical Centre Independent Research Ethics Committee (Ethic Reference Number: SREC 007/2018/FR). All the patients were consented for data and blood sample collection for NGS analysis.

### Panel design

The custom 15-gene ctDNA NGS panel which selectively covered the hotspot regions of 15 clinically significant genes implicated in NSCLC (i.e. *EGFR*, *KRAS*, *NRAS*, *BRAF*, *PIK3CA*, *ERBB2*, *MET*, *KIT*, *PDGFRA*, *ALK*, *ROS1*, *RET*, *NTRK1*, *NTRK2*, *NTRK3*; details as listed in S3 Table) was designed using ArcherDX Assay Designer (Archer, USA). The selected hotspot point mutations or indels serve as predictive biomarkers for targeted therapies approved by the FDA or already listed in the NCCN guidelines.

### Clinical samples

In this pilot study, a total of 10 patients in Sunway Medical Centre with newly diagnosed stage III or IV NSCLC and tested for tissue *EGFR* mutation [*EGFR* positive (n = 6); *EGFR* negative (n = 4)] were recruited into this study. All the blood samples were taken within 1-month from the date of tissue biopsy. A total of 20 ml peripheral blood samples was taken from eligible patients before the initiation of first line treatment. The blood samples were collected into Cell-Free DNA CT^™^ tube (Streck, USA) and processed within 4 hours post-collection. The plasma fraction was separated from the blood cells by two consecutive rounds of centrifugation for 10 min at room temperature, at 1600 g and at 16,000 g, respectively. The plasma fraction (~ 4 ml) was used to isolate ctDNA using the Maxwell RSC LV cfDNA extraction kit (Promega, USA). The ctDNA was eluted with ultra-pure nucleic acid free water (Thermo Fisher Scientific, USA), and stored at -80°C. The purity of ctDNA was determined using Nanodrop (Thermo Fisher Scientific, USA), whereas the concentration and integrity of ctDNA were determined using Quant-iT dsDNA HS Kit (Thermo Fisher Scientific, USA) and DNA 1K Hi Sens LabChip (Perkin Elmer, USA) respectively. Samples which passed quality assessment were subjected to targeted NGS assay.

### Analytical & clinical validation of the custom 15-gene panel

The regions of interest were amplified from 20 ng or 50 ng input ctDNA and the barcode/adaptor was added to each library using Archer MBC Adapters. The libraries were constructed using Archer custom 15-gene lung cancer panel kit according to the manufacturer’s instructions. The concentration of the constructed libraries was assessed using Kapa Library Quantification Kits (Kapa Biosystems, USA) prior to sequencing. For each sequencing run, a range of 12 pM to 16.5 pM libraries were pooled and sequenced on Illumina Miseq using Miseq Reagent Kit V3 (600-cycle). The sequencing run were optimized to achieve the optimum target specification of 1200–1400 k/mm^2^ clusters passing filter as per manufacturer’s instructions. Following this, the mutations were identified using Archer Analysis software (version 6.2).

In order to evaluate the analytical performance of the 15-gene lung cancer panel, a set of 4 multiplex I ctDNA reference standards at different allellic frequencies (0.1%, 1%, 5% and parental) from Horizon Discovery were used for detecting variants in the genes. The covered gene variants were *EGFR* (p.L858R, p.delE746-A750, p.T790M, p.V769-D770insASV), *KRAS* (p.G12D), *NRAS* (p.Q61K, p.A59T) and *PIK3CA* (p.E545K). In addition, Seraseq ctDNA Mutation Mix v2 (1% allelic frequency) was used as reference material for detecting *BRAF* (p.V600E), *EGFR* (p.E746_A750del, p.D770_N771insG, p.L858R, p.T790M), *ERBB2* (p.A775_G776insYVMA), *KIT* (p.D816V), *KRAS* (p.G12D), *NRAS* (p.Q61R), *PDGFRA* (p.D842V, p.S566fs*6), *PIK3CA* (p.E545K, p.H1047R), and *RET* (p.M918T). The analytical performance of the assay was determined by comparing the NGS results with known mutations of the reference standards.

In order to evaluate the clinical performance of the 15-gene lung cancer panel for detecting *EGFR* mutations, only treatment naïve advanced stage III or IV NSCLC patients who were tested for tissue *EGFR* mutations [*EGFR* positive (n = 6), *EGFR* negative (n = 4)] and had ctDNA samples collected within 1-month interval from tissue biopsy were subjected to targeted NGS assay. The performance of the NGS assay in detecting *EGFR* mutations was determined by using tissue *EGFR* result as reference (orthogonal test).

### Bioinformatic analysis

The bioinformatics analysis was performed by using the proprietary Archer Analysis Software Version 6.2 (Archer, USA). The fastq data were uploaded to the cloud and analyzed according to the following settings: alternate observations (AO) ≥ 5, unique alternate observations (UAO) ≥ 3, gnomAD allele frequency ≤ 0.05, variants that have a consequence, variants with an AF outlier p-value ≤ 0.01, and mutant allelic frequency ≥ 0.0055. The variants were confirmed manually with the Integrative Genomics Viewer [28].

## Results

### Patient characteristics

This pilot study has prospectively enrolled a total of 10 patients who were newly diagnosed with advanced stage III or IV lung cancers, and had tested for tissue *EGFR* mutation. The tissue *EGFR* mutations were confirmed via single-gene test [Cobas^®^ EGFR Mutation Test v2 or Therascreen *EGFR* RGQ]. To evaluate the performance of the custom 15-gene ctDNA NGS panel for detecting plasma *EGFR* mutation in clinical setting, peripheral blood samples were collected from these 10 patients prior to the administration of tyrosine kinase inhibitor (*EGFR* positive, n = 6), or other therapies (*EGFR* negative, n = 4). The demographic and clinicopathological characteristics of these patients are summarized in Table 1. Only 1 patient presented with stage III lung cancer, whereas the other 9 patients were diagnosed with stage IV NSCLC. The median age at diagnosis was 66 years (range 55 to 77 years). Majority of the patients were male (n = 7). The average concentration of ctDNA extracted from 4 ml plasma samples was 3.38 ng/μl (range 0.48 ng/μl to 16.6 ng/μl). The amount of ctDNA (60 μl) recovered from each 4 ml sample ranged between 28.68 ng and 996 ng. Based on the amount of recovered ctDNA profiles, 20 ng ctDNA was chosen as the initial input material for evaluation purposes in this study, taking into consideration the need for repeat testing in a real-world setting.

**Table 1:** Demographics and clinical characteristics of 10 lung cancer patients enrolled in this study.

Case	Stage	Smoker	Age	Gender	Tissue EGFR	ctDNA (ng/μl)	Amount ctDNA (ng)
Lung 01	3	Yes	64	Male	Negative	0.48	28.8
Lung 02	4	No	55	Male	Deletion in Exon 19	1.39	83.4
Lung 03	4	Yes	66	Male	Negative	0.63	37.8
Lung 04 (Rep1)	4	No	74	Female	L858R	0.98	58.8
Lung 04 (Rep2)						1.15	69
Lung 05	4	No	60	Male	L858R	1.9	114
Lung 06	4	No	66	Female	Deletion in Exon 19	1.01	60.6
Lung 07	4	No	63	Female	Deletion in Exon 19	0.85	51
Lung 08	4	Yes	72	Male	L858R	0.93	55.8
Lung 09	4	-	77	Male	Negative	11.3	678
Lung 10	4	No	68	Male	Negative	16.6	996

Abbreviations: *EGFR* = epidermal growth factor receptor; ctDNA = circulating tumor DNA; Rep 1 = Replicate 1; Rep 2 = Replicate 2

### Quality metrics of custom 15-gene ctDNA panel

A total of 4 sequencing runs were conducted to evaluate the performance of the custom 15-gene ctDNA panel for detecting the clinically relevant somatic mutations in liquid biopsies. Our results (S1 Table) demonstrated that all the 4 runs did not meet the optimum target specification of 1200–1400 k/mm^2^ clusters passing filter. However, our results showed that increasing library loading concentration from 12 pM to 16.5 pM improved the cluster densities and data output. The clusters passing filter of the four sequencing runs were 711 ± 14 k/mm^2^ (12 pM), 880 ± 20 k/mm^2^ (13 pM), 907 ± 45 k/mm^2^ (15 pM), and 1001 ± 23 k/mm^2^ (16.5 pM). It is noteworthy that data quality met the expected threshold value in which >90% of bases had Phred quality scores above 30.

The reference materials and clinical samples were pooled together in each run to maximize the cost-effectiveness of the testing. A total of 8 samples was sequenced in the first 2 runs, whereas 5 samples were sequenced in the third and fourth run to optimize the assay performance. As shown in S1 Table, an average of 1.7 million mapped reads was recovered from the first 2 runs (8 samples each), whereas an average of 3.3 million mapped reads was recovered in the subsequent 2 runs (5 samples each). The number of mapped reads of each sample varied between 950,516 and 4,604,442, with an average of 95% reads being mapped to target regions.

### Analytical validation

**Fig 1:**
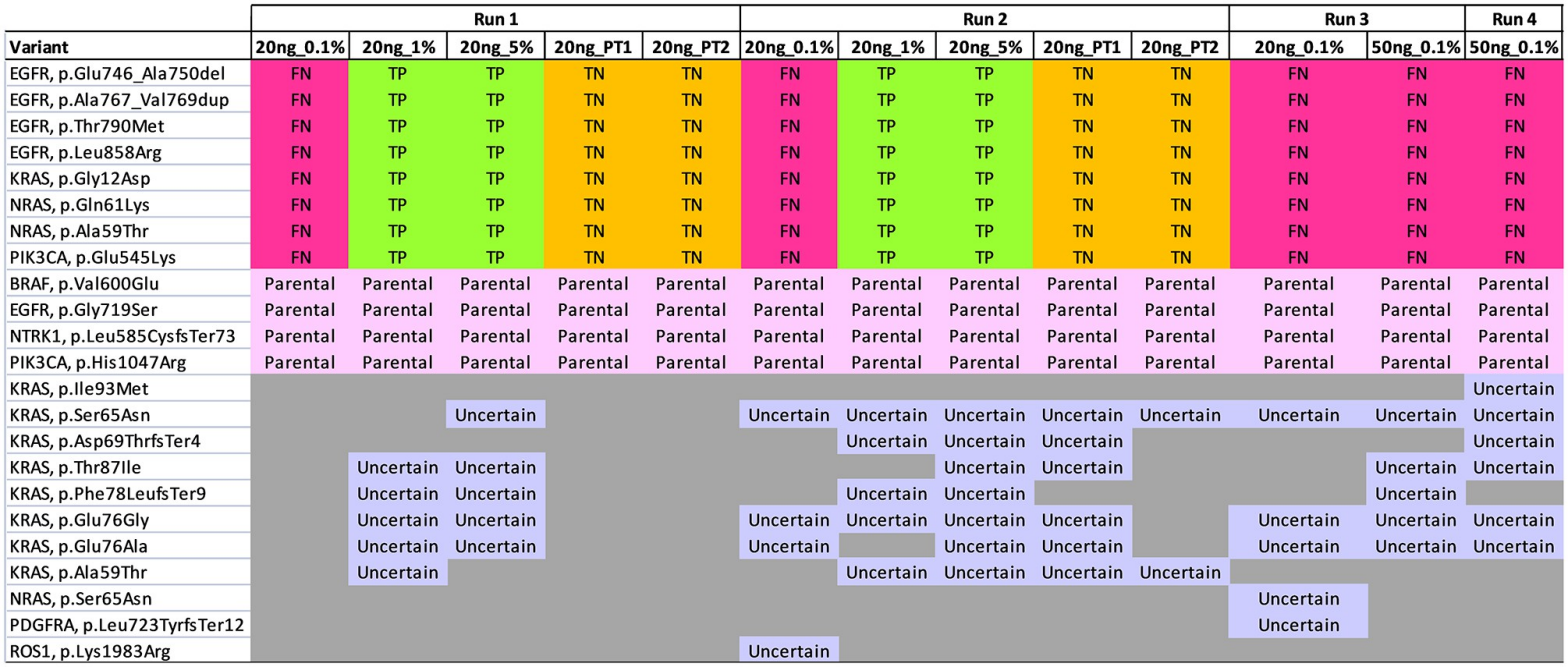
Analytical validation of the Horizon Multiplex I ctDNA reference standards. Variants are listed vertically and samples are shown horizontally. True positive (TP), false negative (FN), true negative (TN), and variants of uncertain significance are shown in green, red, orange and purple respectively. Parental variants are shown in pink (allelic frequencies range 19%–32%). Run 1 and 2 included 20 ng Horizon 5%, 1%, 0.1% and duplicates of parental ctDNA. Run 3 included 20 ng and 50 ng Horizon 0.1% ctDNA. Run 4 included 50 ng Horizon 0.1% ctDNA. Our results demonstrated that the detection limit of the assay was at 1% allelic frequencies, and achieved 100% analytical sensitivity and 100% analytical specificity.

**Fig 2:**
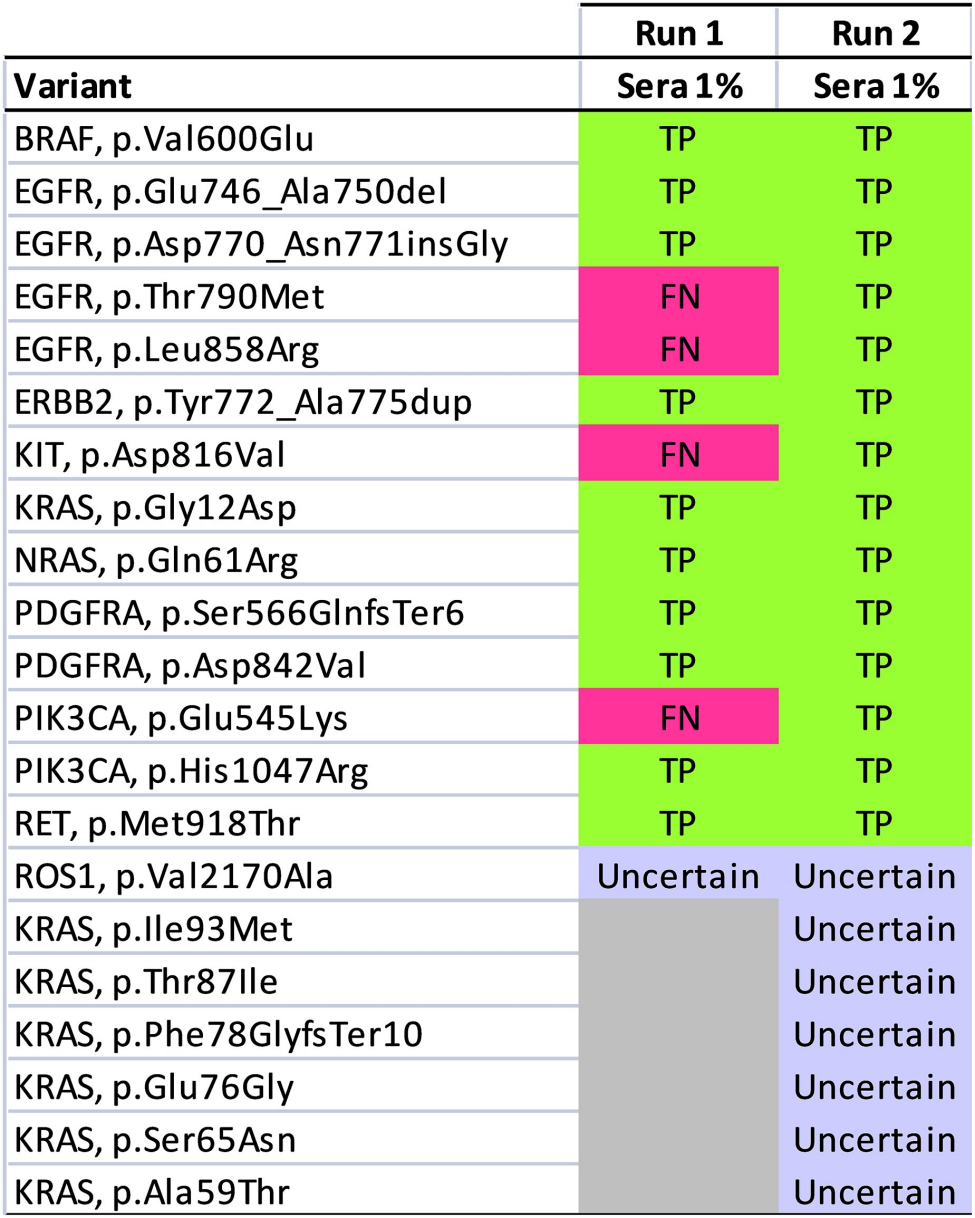
Analytical validation of the Seraseq^®^ctDNA Mutation Mix v2 reference standards. Variants with 1% allelic frequencies are listed vertically and samples are shown horizontally. True positive (TP), false negative (FN), and variants of uncertain significance are shown in green, red and purple respectively. Our results demonstrated that all the known variants were detectable when sequenced to at least ~1.7 million mapped reads.

The analytical performance of the assay was assessed in 4 independent experiments, and the results are summarized in Figs 1 and 2. The details of the variants are listed in S2 Table. Our analysis only focused on known variants covered by the custom panel. The first 2 runs used 20 ng Horizon Multiplex I ctDNA Reference Standards [allelic frequencies: 5% (n = 1); 1% (n = 1); 0.1% (n = 1); parental (n = 2)], and Seraseq^®^ctDNA Mutation Mix v2 [allelic frequency: 1% (n = 1)]. In the first run, a total of 12 pM pooled libraries was sequenced, and yielded a total of 16,872,852 reads passing filter. The 15-gene ctDNA assay detected all the known 8 variants in 5% and 1% Horizon ctDNA, but no variants in 0.1% Horizon ctDNA. All the known variants were not detected in the duplicates of Horizon parental reference standard, giving rise to 100% specificity of these regions. The assay only detected 10 out of 14 known variants in Seraseq^®^ctDNA Mutation Mix v2. The four missing variants were *EGFR* (p.T790M), *EGFR* (p.L858R), *KIT* (p.D816V), and *PIK3CA* (p.Glu545Lys). In the second run, the same set of reference standards were analyzed by increasing the loading concentration to 13 pM, and the run yielded a total of 20,317,542 reads passing filter. Increasing the loading concentration significantly improved the sequencing output. Similar to the performance of first run, the 15-gene ctDNA panel detected all the known 8 variants in 5% and 1% Horizon ctDNA, but failed to detect the presence of variants with 0.1% allelic frequencies. The known variants were absent in the Horizon parental duplicates. The assay detected all the 14 known variants with 1% allelic frequencies in Seraseq^®^ctDNA Mutation Mix v2 when sequenced to ~1.7 million mapped reads.

The input of Horizon 0.1% ctDNA was increased to 50 ng in the third and fourth runs to optimize the detection limit of the assay. In parallel, 20 ng Horizon 0.1% was used in the third run for comparison purposes. The third run was loaded with 15 pM pooled libraries and yielded 21,028,976 reads passing filter. The fourth run was loaded with 16.5 pM and yielded 23,341,198 reads passing filter. Notably, approximately 1.5 million to 2.4 million mapped reads were generated from 20ng of 0.1% Horizon ctDNA, whereas approximately 3.8 million to 4.6 million mapped reads were generated from 50 ng of 0.1% Horizon ctDNA. Our results suggested that the increase of input and throughput of 0.1% Horizon ctDNA did not improve the detection limit of the assay whereby all the known variants in 0.1% Horizon ctDNA were not detected.

Our analysis consistently detected four variants in the Horizon Multiplex I cfDNA Reference Standards which were of parental cell line origin. The allelic frequencies were ~30% in *BRAF* p.Val600Glu, ~20% in *EGFR* p.Gly719Ser, ~25% in *NTRK1* p.Leu585CysfsTer73, and ~23% in *PIK3CA* p.His1047Arg. In addition, the combined analysis of these 4 independent runs revealed that this panel may not be suitable for reporting the following variants which are randomly detected in the samples, including *KRAS* (p.Ile93Met, p.Ser65Asn, p.Asp69ThrfsTer4, p.Thr87Ile, p.Phe78LeufsTer9, p.Glu76Gly, p.Glu76Ala, p.Ala59Thr), *NRAS* (p.Ser65Asn), *PDGFRA* (p.Leu723TyrfsTer12), and *ROS1* (p.Lys1983Arg, p.Val2170Ala).

Overall, our analysis demonstrated that the detection limit of the custom 15-gene ctDNA assay was at 1% allelic frequency. The assay achieved 100% analytical sensitivity and 100% specificity when 20 ng ctDNA was sequenced to at least ~1.7 million mapped reads.

### Clinical validation

To determine the concordance between plasma and tissue *EGFR* variants, the peripheral blood samples were taken within 1-month interval from the date of the tissue biopsies. A total of 10 patients with newly diagnosed advanced stage III or IV lung cancer were recruited into this study. These clinical samples were sequenced concurrently with analytical reference standards in 4 independent runs. A total of 4 clinical samples was tested by using 20 ng input, whereas 7 samples were tested by using 50 ng input ctDNA. As shown in S1 Table, an average of ~2.7 million mapped reads were recovered from the clinical samples [range 1,354,134 to 3,628,524 mapped reads].

**Fig 3:**
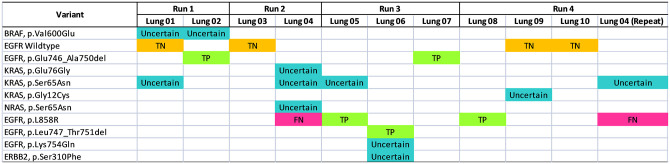
Clinical validation of the 10 patients with newly diagnosed advanced stage III or stage IV lung cancer. Variants are listed vertically and samples are shown horizontally. Known *EGFR* true positive (TP), true negative (TN) and false negative (FN) are shown in green, orange and red respectively. Comparison with tissue *EGFR* demonstrated that the 15-gene ctDNA panel achieved 83.3% clinical sensitivity and 100% clinical specificity. In addition to known *EGFR* mutation status, the NGS assay detected additional variants which are shown in blue, and confirmation with orthogonal method is warranted to confirm its accuracy.

The clinical performance of the assay was assessed in 4 independent experiments, and the results are summarized in Fig 3. The details of the variants are listed in S2 Table. In comparison with tissue *EGFR*, the 15-gene ctDNA assay detected 5 out of 6 known positive variants, and 4 out of 4 known negative variants, giving rise to 83.3% clinical sensitivity and 100% clinical specificity. The discordant result was observed in Lung 04 which harbored *EGFR* p.L858R mutation. The circulating *EGFR* variant remains undetectable even though the experiment on Lung 04 was repeated by increasing the ctDNA input from 20 ng which yielded 1,778,994 mapped reads to 50 ng which yielded 3,053,604 mapped reads. We postulated that the discordance could be attributed to the low abundance of circulating *EGFR* mutation during sampling time, and was below the detection limit of the assay.

Other than known tissue *EGFR* variants, the assay also detected additional variants in these 10 clinical samples. *BRAF* p.Val600Glu was found in Lung 01 and Lung 02, while *KRAS* p.Gly12Cys was found in Lung 09. *EGFR* p.Lys754Gln and *ERBB2* p.Ser310Phe were found in Lung 06. *KRAS* p.Ser65Asn was detected in Lung 01, duplicates of Lung 04 and Lung 05. Also, *KRAS* p.Glu76Gly and *NRAS* p.Ser65Asn were detected in Lung 04.

Overall, our analysis demonstrated that the custom 15-gene ctDNA panel achieved 83.3% clinical sensitivity and 100% specificity for detecting *EGFR* variants. The usefulness of the panel in detecting other variants remains unknown and confirmation via orthogonal method is required for low confidence variants which were randomly detected in the tested samples.

## Discussion

With the list of actionable mutations for solid cancers steadily expanding, the development of NGS panel for the simultaneous analysis of actionable targets in liquid biopsy is desirable. In year 2020, the approval of Guardant360 CDx test (Guardant Health) and FoundationOne Liquid CDx test (Foundation Medicine, Inc.) as the first two liquid biopsy NGS tests for advanced solid cancers by the FDA marked a new breakthrough in the field of non-invasive molecular testing. Over the years, numerous studies have been conducted to evaluate the performance of custom NGS-based ctDNA panels, with some showing promising results. For instance, Digital Sequencing^™^ demonstrated analytical sensitivity down to 0.1% mutant allele fraction, and clinical sensitivity and specificity of 85%, and 99.6% respectively as compared to tissue-based NGS [29]. Firefly assay demonstrated superior sensitivity and specificity with a 98.89% detection rate at an allele frequency of 0.2% [30]. Oncomine^™^ Lung cell-free DNA Assay (OLcfA) NGS panel has been shown to be effective in detecting mutations in NSCLC patients with a detection limit of 0.1% [31]. In this proof-of-concept pilot study, we aim to develop and validate a custom 15-gene ctDNA NGS panel for detecting single nucleotide variants and small indels in lung cancer, with primary focus on *EGFR* mutation analysis in NSCLC. The panel harnessed molecular barcoding technology which aims to differentiate the true variants from PCR duplicates and arising artifacts by taking into consideration unique DNA fragments counts [32, 33].

*EGFR* mutations represent the most commonly detected genetic alteration in NSCLC, with a prevalence range of 40–60% among Asians [34–36]. NSCLC patients who received epidermal growth factor receptor tyrosine kinase inhibitors (TKI) demonstrated excellent responses and achieved longer progression free survival [37, 38]. Testing of tissue *EGFR* mutations is the gold standard to guide TKI treatment in NSCLC. In the event that tissue sample is inadequate or inaccessible, plasma *EGFR* test is the prioritized option. Clinically, Cobas *EGFR* Mutation Test v2, and Therascreen^®^ *EGFR* Plasma RGQ are the two FDA approved real-time PCR liquid biopsy companion diagnostic assay for the detection of plasma *EGFR* mutations in NSCLC. The reported detection limit of Cobas and Therascreen for most common *EGFR* mutations ranged between 1.3–13.4%, and 0.81–17.5% respectively. Subsequently, droplet digital PCR with lower detection limit (0.1% allelic frequency) has been developed and widely used as a monitoring tool for patients receiving EGFR inhibitors and enabling rapid switch to third-generation TKI in patients who have acquired the T790M mutation [39–41]. Overall, our custom NGS panel showed comparable performance to the real time PCR based assays (Cobas and Therascreen) and achieved 100% analytical sensitivity and specificity for detecting EGFR mutations with at least 1% allelic frequencies. However, the assay is suboptimal when compared to droplet digital PCR or NGS-based assays which can achieve detection limits of below 1% [18, 31, 41].

Clinical evaluation of *EGFR* mutations showed that our NGS assay achieved 83.3% sensitivity and 100% specificity, in which *EGFR* p.L858R was missed in 1 out of 6 confirmed cases. We speculate that the discordance could be attributed to several inherent limitations of ctDNA, such as short half-life of <1.5 hours and low abundance of ctDNA relative to normal cell free DNA. Also, technical challenges such as the amount of input ctDNA, sequencing reads depth, and bioinformatics algorithms could be the key extrinsic factors that affect the assay performance [11, 13, 42, 43]. An extension study is required to further optimize and assess the clinical utility of this panel.

In addition to *EGFR*, we detected several other mutations, including *BRAF*, *ERBB2*, *NRAS*, and *KRAS* in 6 out of 10 clinical samples. We recognized the lack of balance ctDNA for further confirmation testing via orthogonal methods in the present study. Nonetheless, the assay can detect all of the known mutations in the reference samples when adequately sequenced. Increasing evidences have shown that the co-existence of *EGFR* with *KRAS*, *BRAF*, and *ERBB2* are not uncommon, and were associated with resistance to EGFR inhibitors and poorer survival [44–52]. These concomitant mutations were detected in nearly 70% of *EGFR* positive cases of this study [*BRAF* (1/6); *KRAS* (2/6), *ERBB2* (1/6)], and their therapeutic impacts remain to be investigated. Multigene testing is advantageous in providing a more comprehensive genetic information for guiding treatment decision. *BRAF*, *ERBB2* and *RET* have molecular targeted drugs available for use as therapy. Therefore in 30% of patients, liquid biopsy identified additional therapies that would not have been detected on standard molecular testing. Within this context, Lung 06 may be more amenable to afatinib, evidenced by the success of afatinib in treating metastatic lung adenocarcinoma harboring *EGFR* (p.L858R) and *ERBB2* (p.S310F) co-mutations [53]. Lung 02 could be treated with BRAF inhibitors as a rescue regimen to EGFR inhibitors, hence broadening the therapeutic options. Multigene panel testing can therefore potentially provide a powerful tool in delivering precision oncology, especially in patients with refractory diseases.

## Conclusion

Overall, our analysis demonstrated that the limit of detection for the 15-gene ctDNA NGS panel for EGFR variants was at 1%, and is comparable to other PCR based methods, including Cobas^®^ EGFR Mutation Test v2 and Therascreen EGFR plasma RGQ. The assay achieved 100% analytical sensitivity and specificity when sequenced to at least ~1.7 million mapped reads. The clinical sensitivity and specificity of the assay for detecting *EGFR* mutations were 83.3% and 100% respectively. Our preliminary analysis suggests that the custom NGS assay is of good performance. Extension studies are warranted in order to optimize the assay and to include validation of other candidate gene mutations.

## Supporting information

S1 Table Summary of sequencing metrics of 4 independent runs by using Illumina MiSeq Reagent Kit v3 (600-cycle). (XLSX)

S2 Table Summary of the variants detected by custom 15-gene ctDNA panel. The analysis was performed by Archer Analysis software.
(XLSX)

S3 Table List of regions covered in custom 15-gene ctDNA panel. (XLSX)
